# Biological Activity and Photostability of Biflorin Micellar Nanostructures

**DOI:** 10.3390/molecules20058595

**Published:** 2015-05-13

**Authors:** Edson R. B. Santana, João P. Ferreira-Neto, Ricardo Yara, Kêsia X. F. R. Sena, Adriana Fontes, Cláudia S. A. Lima

**Affiliations:** 1 Departamento de Biofísica e Radiobiologia, Universidade Federal de Pernambuco, Recife, PE 50670-901, Brazil; E-Mails: erbs2k@gmail.com (E.R.B.S.); joaopaulo.ferreiraneto@gmail.com (J.P.F.-N.); 2 Departamento de Engenharia Biomédica, Universidade Federal de Pernambuco, Recife, PE 50670-901, Brazil; E-Mail: ricardo.yara@ufpe.br; 3 Departamento de Antibióticos, Universidade Federal de Pernambuco, Recife, PE 50670-901, Brazil; E-Mail: k.xisto@gmail.com

**Keywords:** biflorin, biological activity, micellar nanostructures, photostability, stability

## Abstract

*Capraria biflora* L. is a shrub from the Scrophulariaceae family which produces in its roots a compound named biflorin, an *o*-naphthoquinone that shows activity against Gram-positive bacteria and fungi and also presents antitumor and antimetastatic activities. However, biflorin is hydrophobic and photosensitive. These properties make its application difficult. In this work we prepared biflorin micellar nanostructures looking for a more effective vehiculation and better preservation of the biological activity. Biflorin was obtained, purified and characterized by UV-Vis, infrared (IR) and ^1^H- and ^13^C-NMR. Micellar nanostructures of biflorin were then assembled with Tween 80^®^, Tween 20^®^ and saline (0.9%) and characterized by UV-Vis spectroscopy and dynamic light scattering (DLS). The results showed that the micellar nanostructures were stable and presented an average size of 8.3 nm. Biflorin micellar nanostructures’ photodegradation was evaluated in comparison with biflorin in ethanol. Results showed that the biflorin in micellar nanostructures was better protected from light than biflorin dissolved in ethanol, and also indicated that biflorin in micelles were efficient against Gram-positive bacteria and yeast species. In conclusion, the results showed that the micellar nanostructures could ensure the maintenance of the biological activity of biflorin, conferring photoprotection. Moreover, biflorin vehiculation in aqueous media was improved, favoring its applicability in biological systems.

## 1. Introduction

*Capraria biflora* L. is a perennial shrub native to the Antilles and South America that belongs to Schrophulariaceae family [1,2,3]. This species has been employed in a variety of reported folk medicine applications as a diuretic, stimulant, gastrointestinal agent, and analgesic, among other phytotherapeutical uses [4,5,6]. In the 1950s, a violet substance, that was denominated biflorin, was isolated from the roots of *C. biflora* L. [7]. Biflorin [6,9-dimethyl-3-(4-methyl-3-pentenyl)naphtha[1,8-bc]-pyran-7,8-dione] is an prenylated *o*-naphthoquinone, which can present activity against Gram-positive and alcohol-acid resistant bacteria and filamentous fungi [8]. In addition to its antimicrobial activity, recent studies have indicated that biflorin can be used to inhibit the growth of some carcinogen cell lines [3], mainly melanoma cells [9]. Besides, some authors have also reported that biflorin can present immunoadjuvant properties [10] and antimetastatic activity, also for melanoma cells [11]. Moreover, studies of biflorin cytotoxicity showed that this molecule did not cause any damage to V79 cells (hamster lung fibroblasts) and did not present any negative interference with the development of sea urchin eggs [3,12].

A few years after its first use, studies with biflorin have reported that this substance is photodegradable [13]. The authors also showed that the biological activity of the biflorin can be drastically decreased after light exposure [13], which can hinder the use and manipulation of this molecule without the loss of part of its activity. It was also reported that the biological activity of biflorin against dermatophyte fungi decreased around 50% after light exposure [14]. Studies like those indicate that the photosensitivity, as well as the hydrophobic characteristics of the biflorin, can be relevant factors which can make difficult the utilization of this molecule as a pharmaceutical agent for biological applications. Therefore novel vehiculation forms of this substance should be used as an alternative to improve its use. Pharmaceutical forms like polymeric nanoparticles, emulsions, and others, may provide physical barriers that can avoid or, at least, considerably decrease the effects of photodegradation on photosensitive molecules [15].

In this context, the aim of this study was to formulate and characterize biflorin micellar nanostructures in order to preserve this molecule from photodegradation and also improve the biocompatibility of this substance. We also tested the biological activity of the biflorin micellar nanostructures against Gram-positive and Gram-negative bacteria and yeasts. All experiments were performed in comparison with biflorin in ethanol and/or dimethyl sulfoxide (DMSO), since these solvents have been usually employed in the biological tests reported in the literature [8,9,10,11,12,13,14]. Compared to these previous studies, we believe that the use of micellar nanostructures can improve the biflorin vehiculation in aqueous media expanding its biological applications and prevent, or at least reduce its photodegradation, ensuring the maintenance of the biological activity of this molecule, even after a long period of exposure to light.

## 2. Results and Discussion

### 2.1. Biflorin Micellar Nanostructures Characterization and Stability

**Figure 1:**
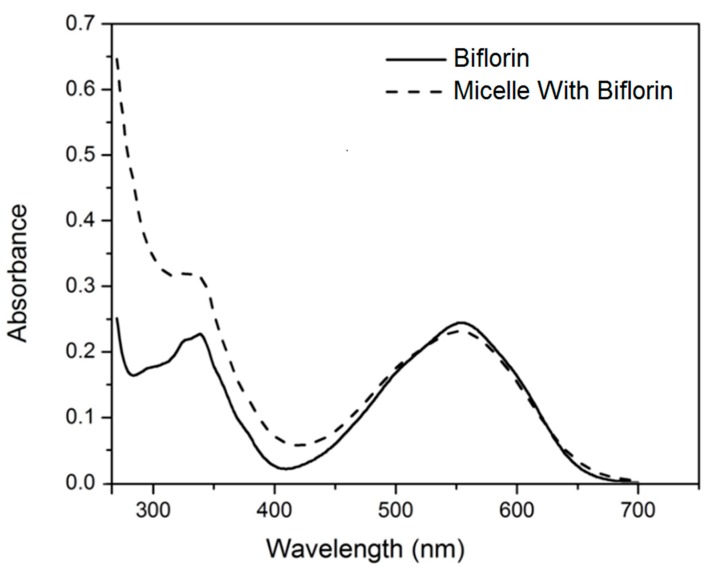
Absorption spectra of biflorin in ethanol (solid line) and biflorin micellar nanostructures (dashed line).

UV-Vis, IR and NMR analysis results confirmed the biflorin structure and matched the corresponding literature data [14,16]. Typical bands were observed at λ_max_ 340 and 555 nm in the UV-Vis analysis. The biflorin micellar nanostructures presented a translucent violet aspect. The absorption spectrum of biflorin micellar nanostructures, in comparison with the biflorin in ethanol (Figure 1), showed the same typical maximum band around 555 nm, confirming the presence of this molecule in the formulation. The band around 340 nm also was present, but it was more accentuated for the biflorin micellar nanostructures due to the typical surfactant absorption bands in the UV region.

**Figure 2:**
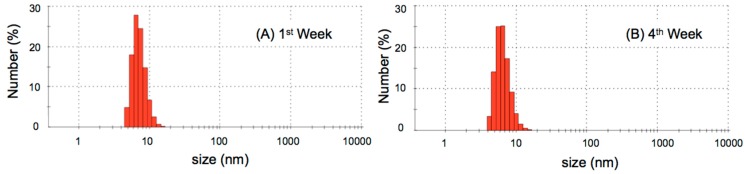
Typical DLS measurements for biflorin micellar structures during the first week (**A**) and fourth week (**B**), after preparation (Treatment A).

Dynamic light scattering analyzes were done one day per week during one month. Average sizes and polidispersion index (PDI) for the micellar nanostructures from Treatment A (4 °C) and Treatment B (room temperature) are presented in Table 1. Droplet sizes, as well as PDI values, for the Treatment A samples did not present any significant changes, confirming the stability of the formulation during the analysis period. The biflorin micellar nanostructures from Treatment A showed a constant average size around 8.3 nm during the whole month. Typical dynamic light scattering plots of biflorin micellar structures during the first week (A) and the fourth week (B), for the Treatment A, are presented in Figure 2. On the other hand, the droplet sizes of samples, after Treatment B, increased substantially over time and PDI values were always high, showing a destabilization process for these micellar nanostructures as a consequence of the higher storage temperature [17].

**Table 1:** Average size and PDI values as a function of time for biflorin micellar nanostructures stored under different temperature conditions.

Treatment A			Treatment B	
Week	Size (nm)	PDI	Size (nm)	PDI
1	8.2 ± 0.1	0.06 ± 0.01	8.2 ± 0.5	0.05 ± 0.01
2	8.3 ± 0.1	0.06 ± 0.01	46.9 ± 18.0	0.30 ± 0.01
3	8.4 ± 1.1	0.08 ± 0.01	57.0 ± 15.5	0.30 ± 0.01
4	8.2 ± 0.7	0.08 ± 0.01	96.4 ± 10.2	0.33 ± 0.01

In addition, the UV-Vis analysis, even for the samples of treatment B, did not indicate changes in the absorbance values, suggesting that precipitation of the biflorin did not occur. The absorbance values obtained for four weeks of analysis, measured at 555 nm, were approximately: 0.26 (1st week), 0.26 (2nd week), 0.27 (3rd week) and 0.29 (4th week). As observed, the absorbance did not change significantly as a function of time. Only a slight increase of the values was observed, probably due to a possible higher scattering caused by the increased droplet size.

Thus, we can conclude that the micellar nanostructure absorbances, as well as the properties of the biflorin, were preserved for both treatments. In this way, despite the lower stability of the micellar nanostructures with Treatment B, the biflorin characteristics were maintained and it still could be viable for applications when the droplet size was not a crucial factor.

The stability of the formulation after centrifugation, heating and cooling was evaluated by DLS analysis. The hydrodynamic sizes found were: (a) Control: (9.6 ± 0.2) nm with PDI (0.07 ± 0.01); (b) after heating, (10.1 ± 0.1) nm with PDI (0.09 ± 0.01); (c) after cooling, (9.5 ± 0.2) nm with PDI (0.08 ± 0.01) and (d) after centrifugation, (9.8 ± 0.2) nm with PDI (0.08 ± 0.02). As one can observe, only after heating, a slightly higher formulation’ size were observed, approximately 5% larger than control. Slight differences in sizes were also observed in this last experiment, when compared to previous ones, due to the purification and the fast crystallization methods used to obtain biflorin for these stability experiments. These results confirm a homogeneous distribution of small droplets for the micelles and the stability of the formulation, as described in earlier works on oil-in-water systems [18,19].

The results also showed that approximately 97% of the biflorin were encapsulated. The absorbance values found before (as prepared) and after (centrifugation/filtration) at the biflorin maximum band (555 nm) were about: 0.284 (20 µg/mL) and 0.275 (19.4 µg/mL).

### 2.2. Photostability Experiment

**Figure 3:**
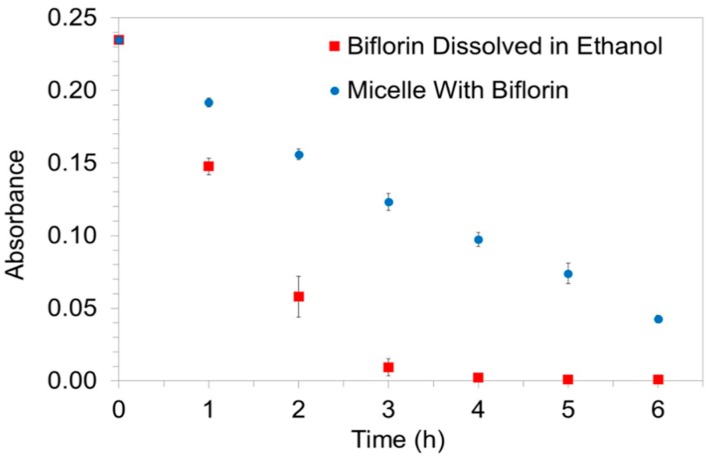
Biflorin micellar nanostructure (blue dots) and biflorin in ethanol (red squares), absorbance at 555 nm evaluated in function of the exposure time in a photostability chamber. Error bars represents standard deviation.

The degradation of the biflorin present in the micellar nanostructures, as well as dissolved in ethanol, was verified by UV-Vis spectroscopy analysis (Figure 3). The plot of Figure 3 was constructed by using the changes in the biflorin absorbance maximum at 555 nm, but we have observed that the other maximum also varied in the same way. After two hours of exposure, the degradation of biflorin dissolved in ethanol quickly reached critical levels in contrast to the biflorin in micellar nanostructures, for which the degradation reached approximately the same level only after six hours of exposure.

According to the literature, one hour of light exposure in this photostability chamber is equivalent to approximately ten hours in a naturally well-lit environment [20]. Therefore, we can conclude that micellar nanostructures can provide around three times more of photoprotection to biflorin than ethanol solutions. Besides, micellar nanostructures can receive natural light directly exposure for around 60 h before complete degradation.

Our results are in accordance with previous ones, which also used pharmaceutical formulations for the improvement of the photostability of drugs [21,22]. For example, Lai *et al.*, showed that nanoemulsions protected tretinoin from UV degradation when compared to tretinoin dissolved in methanol [21]. Our data also corroborated results obtained with rutin, which showed that nanoemulsions of this substance have better photostability than rutin in ethanol [22].

### 2.3. Microbiological Activity

The efficiency of biflorin micellar nanostructures, as well as biflorin in DMSO (10%), against Gram-positive bacteria was confirmed, since the MIC values for these microorganisms were lower than 3.12 µg/mL, the minimal concentration used in the test (Table 2). For yeasts, the inhibition only occurred at high concentrations of biflorin, around 50 µg/mL for micellar nanostructures and 100 µg/mL for the DMSO (10%). This result showed an improvement of the antimicrobial activity against yeasts when the biflorin was vehiculated as micellar nanostructures (Table 2). For Gram-negative bacteria, even at the highest concentrations tested, the biflorin did not present activity in any of the forms of vehiculation applied (Table 2). It is interesting to note that the biflorin was not completely soluble in DMSO (10%), in contrast to previous works [9,10]. Actually, we observed some biflorin precipitation in these samples. In general, these microbiological results are in accordance with previous data, where biflorin was effective against Gram-positive bacteria, with MIC values similar to ours, and did not show any relevant effect against Gram-negative bacteria. For yeasts, results reported in the literature are variable, changing according to the species and, in some cases, with the strain that was used [7,8,14].

**Table 2:** MIC values for biflorin micellar nanostructures and biflorin dissolved in DMSO.

Microorganism	Biflorin Micellar Nanostructures (µg/mL)	Biflorin in DMSO (µg/mL)
*Staphylococcus aureus*	3.12	3.12
*Micrococcus luteus*	3.12	3.12
*Bacillus subtilis*	3.12	3.12
*Escherichia coli*	>100	>100
*Candida krusei*	50	100
*Candida albicans*	50	>100

## 3. Experimental Section

### 3.1. Biflorin Extraction, Purification and Characterization

Biflorin was obtained from roots of *C. biflora* L. cultivated in the Laboratory of Biophysical Chemistry (LBQ), Biological Sciences Center (CCB), Federal University of Pernambuco (UFPE). A voucher specimen (70,028) was deposited at the Herbarium UFPE—Geraldo Mariz, Department of Botany, Biological Sciences Center (CCB), Federal University of Pernambuco (UFPE). Roots were collected and dried in an oven under forced-air circulation at 45 °C during 48 h. Then, they were powdered and extracted, thrice, with 70% ethanol and an ethanolic crude extract was thus obtained. The extract was filtered and evaporated under vacuum at 45 °C for solvent removal. The residue was chromatographed over silica gel (230–400 mesh), using gradient mixtures of toluene and ethyl acetate as eluents. The obtained fractions were grouped according to results obtained from thin layer chromatography (TLC) analysis. Then, the fractions containing biflorin were distilled in a rotary evaporator and the residue was recrystallized with diisopropyl ether. After this, the purified crystals of biflorin were collected and dried. The biflorin was characterized by the conventional spectroscopic methods UV-Vis (UV-1800, Shimadzu, Kyoto, Japan), infrared spectroscopy (IFS 66, Bruker, Billerica, MA, USA) and ^1^H- and ^13^C-NMR (Varian Unity plus 300 MHz, Garden State Scientific, Somerville, NJ, USA), and the data were compared with the literature [14,16].

### 3.2. Micellar Nanostructure Formulation and Characterization

Micellar nanostructures containing biflorin were prepared with 265 mg of Polysorbate 80 (polyoxyethylene (20) sorbitan monooleate, Tween 80^®^, Vetec/Sigma-Aldrich, St. Louis, MO, USA) and 265 mg of Polysorbate 20 (polyethylene glycol sorbitan monolaurate, Tween 20^®^, Vetec/Sigma-Aldrich, St. Louis, MO, USA) as surfactants. The dispersed phase was composed by biflorin itself (5 mg) and the continuous phase was consisted of 0.9% saline solution. The final volume of 250 mL resulted in a concentration of approximately 20 µg/mL. Biflorin crystals and surfactants were mixed by manual agitation with a glass rod under heating up to 70 °C. Then, saline solution was added and the micellar nanostructures were finished under sonication for 20 minutes in an ultrasound bath (Ultra Cleaner 1400 A, Unique, Indaiatuba, Brazil) at a frequency of 25 kHz. In order to verify the stability of formulations with time, samples of micellar nanostructures, separated into two different treatments, were analyzed in duplicate. The samples of Treatment A were stored at 4 °C. For Treatment B, samples were stored at room temperature (*ca.* 25 °C). Both treatments were protected from the light.

Weekly analyzes by UV-Vis spectroscopy (UV-1800) and dynamic light scattering (DLS) (Zetasizer Nano ZS 90, Malvern, Worcestershire, UK) were performed during one month. Tests of stability, based on DLS analysis, were also performed by evaluating the hydrodynamic sizes after centrifugation (6600× *g*), heating (*ca*. 60 °C) and cooling (*ca.* −6 °C) the formulation. For stability experiments, new amounts of biflorin were obtained by flash chromatography using an Isolera One Flash Purification System (Biotage, Uppsala, Sweden) and SNAP Ultra pre-packed cartridges (25 g, Biotage, using toluene-EtOAc, 9:1 to 7:3, v/v). The isolated biflorin was crystallized using diisopropyl ether [23].

For determining the encapsulation efficiency of biflorin in the micellar nanostructures, first the absorbance of micelles containing biflorin (as prepared) was evaluated by UV-Vis analysis (at 555 nm). After, the nanosystems were centrifuged at 6600× *g* and the absorbance of the supernatant, after filtration by a PES membrane syringe-filter (0.22 µm), was also evaluated. Thus, by analyzing the differences in UV-Vis absorbance of the formulation before (as prepared) and after the centrifugation/filtration, it was possible to estimate the encapsulation efficiency [24,25].

### 3.3. Photodegradation Study

According to the literature [13], biflorin is a photodegradable substance and this characteristic can decrease its biological activity against microorganisms after the light exposure. Therefore, we evaluated the photodegradation of biflorin as an ethanolic solution and as micellar nanostructures. Samples of biflorin micellar nanostructures and biflorin in ethanol 92.8° INPM (Santa Cruz, Guarulhos, Brazil), both at 20 µg/mL, were placed in a photostability chamber. Samples of quinine monohydrochloride dehydrate (Sigma-Aldrich, St. Louis, MO, USA) at 2% were used as positive control for the photodegradation analysis. This procedure was performed according to Brazilian Health Surveillance Agency (ANVISA) parameters for photostability studies [26]. The photostability chamber used was equipped with six white fluorescent lamps (58 Watts) and two UV lamps (80 Watts), and was previously calibrated [20]. The experiment was carried out with samples under over six hours of light exposure. The test comprised seven flasks for each sample type: biflorin micellar nanostructures, biflorin in ethanol and quinine. One flask from each sample were kept outside the chamber and protected from the light (control group). The other six flasks were maintained inside the chamber and removed one by one after each one hour of interval until it was completed six hours of experiment. All treatments were performed in triplicate. Samples were then analyzed by UV-Vis spectroscopy and the absorbance spectra were used to evaluate the photodegradation.

### 3.4. Biological Assays

The efficiency of biflorin micellar nanostructures against microorganisms was evaluated, in comparison with biflorin in dimethyl sulfoxide (DMSO, Vetec/Sigma-Aldrich, St. Louis, MO, USA), and was performed using minimum inhibitory concentration (MIC) analysis. The experiment included biflorin micellar nanostructures and biflorin in DMSO, in triplicate. DMSO was used instead of ethanol because this solvent is usually applied in biological activity experiments and it would allow a comparison with other authors’ data [3,4,5,6,7,8,9,10,11]. Microorganisms used in the test were *Staphylococcus aureus* UFPEDA01, *Bacillus subtilis* UFPEDA16, *Micrococcus luteus* UFPEDA06 (Gram-positive), *Escherichia coli* UFPEDA224 (Gram-negative), *Candida albicans* UFPEDA1009 and *C. krusei* UFPEDA1002 (yeasts). Bacteria were cultured in Mueller Hinton medium and yeasts in Sabouraud medium. The assay was performed with samples of biflorin in DMSO and as micellar nanostructures, both were filtered using 0.22 µm membrane filters (Techno Plastic Products, Trasadingen, Swiss) and distributed by serial dilution in the 96-well plates. Each well contained 10^7^ microorganisms with the respective culture media, resulting in a final volume of 100 µL per well. The concentrations of biflorin, for both types of formulation employed in the tests were 100 µg/mL, 50 µg/mL, 25 µg/mL, 12.5 µg/mL, 6.25 µg/mL and 3.12 µg/mL. Micellar nanostructures components and DMSO (10%) without biflorin were used as negative control. All procedures were performed under sterile conditions in a laminar flow chamber.

## 4. Conclusions

The characterization of biflorin micellar nanostructures showed that this formulation was stable for at least one month, when stored under appropriate temperature conditions. We also showed that the photoprotection, conferred by the micellar nanostructures to biflorin, reduces the degradation of the molecule and preserves its activity. The antimicrobial activity was maintained, or slightly improved, for all the microorganisms tested. Therefore, we conclude that micellar nanostructures can be a good pharmaceutical formulation for biflorin vehiculation, which will ensure its preservation even after light exposure and will enhance the distribution of this substance in an aqueous medium.
